# The Clinicopathological Comparison of Diagnostic Curettage and Total Abdominal Hysterectomy Results in Patients with Endometrial Cancer Referred to Shahid Sadoughi Hospital, Yazd, During the Years 2019 To 2023: The Diagnostic Accuracy of Curettage in Endometrial Cancer

**DOI:** 10.31661/gmj.v14i.3736

**Published:** 2025-03-04

**Authors:** Fahimeh Nokhostin, Fatemeh Mazidi, Maryam Vajihinejad

**Affiliations:** ^1^ Department of Obstetrics and Gynecology, Faculty of Medicine, Shahid Sadughi University of Medical Sciences, Yazd, Iran; ^2^ Department of Pathology, Shahid Sadoughi Hospital, Shahid Sadoughi University of Medical Sciences,Yazd,Iran

**Keywords:** Endometrial Neoplasms, Curettage, Hysterectomy, Data Accuracy

## Abstract

**Background:**

The use of curettage in diagnosing endometrial disorders the most common diagnostic method before performing hysterectomy. The previous studies showed that curettage is an inadequate diagnostic method for focal uterine lesions but has high diagnostic accuracy in endometrial hyperplasia and carcinoma. The objective of the present study is to compare the clinicopathological results of diagnostic curettage and total abdominal hysterectomy in patients with endometrial cancer.

**Materials and Methods:**

This cross-sectional analytical study was conducted on 85 women with endometrial cancer; 19 high and 66 with low tumor grade between 21.3.2019 and 19.03.2024 who underwent diagnostic curettage and total abdominal. All reports related to pathology samples were extracted according to the inclusion and exclusion criteria of the study. All the information required for the study including the pathological result of diagnostic curettage, the pathological result of a total abdominal hysterectomy, tumor grade was extracted. the statistical tests of Fisher’s Exact Test, Chi-Square Tests were used in SPSS 22.0 software; the significance level was considered equal to 0.05. Furthermore, sensitivity, specificity, positive predictive value, negative predictive value and Kappa coefficient were obtained.

**Results:**

The mean age of the studied samples was 58.825 ± 11.324 (33-90) years. The most common clinical manifestations in the participants were reported postmenopausal bleeding with a frequency of (49.4%) for 42 patients and abnormal uterine bleeding with a frequency of (41.2%) for 35 patients, respectively. The sensitivity, specificity, positive and negative predictive values calculated for curettage were 84.21%, 83.33%, 59.25% and 94.82%, respectively and the kappa coefficient was reported equal to 0.587. it can be said that the results obtained from histopathology of curettage and hysterectomy are consistent (P-value=0.001).

**Conclusion:**

Therefore, in the present study it is concluded that the diagnostic accuracy of curettage, compared to total hysterectomy, is a relatively suitable diagnostic method for evaluating endometrial cancer; this method may be used in the timely diagnosis of this disease. However, caution is required due to the lower PPV, necessitating further evaluation before definitive management.

## Introduction

The incidence of endometrial carcinoma is increasing compared to cervical carcinoma; however, the rate of this ratio is varied in different countries [1]. During the past few decades, endometrial cancer has been the most common malignancy of the female reproductive tract in the United States [2]. This disease is included in 6% of all female cancers worldwide and 2% of all female cancers in Iran [3]. In developing countries, endometrial cancer is the second most common cancer after cervical cancer [4]. The age-incidence curve for endometrial cancer shows that the highest prevalence of diagnosis is in the seventh decade of life [5]. According to the estimations, 4.6 women among 100,000 women in the United States die from uterine cancer. These statistics indicate that uterine cancer poses a great burden on the health care system and timely prevention, diagnosis, and treatment can lead to improved patient outcomes and also a reduction in the burden of the disease [6]. Endometrial carcinoma is divided into two major categories, demographically and histopathologically. The first type is endometrioid carcinoma, which develops in conditions of increased estrogen, such as polycystic ovary syndrome, estrogen-producing ovarian tumors, obesity, and nulliparity. The second type, which is less common, is seen in older ages and is not associated with increased estrogen [7]. In terms of clinical symptoms, in 91% of patients, symptoms appear in the form of postmenopausal bleeding or irregular bleeding, and other symptoms, such as abdominal pain, are less common [8]. The prognosis of endometrial carcinoma in well- and moderately differentiated tumors and are in early stages is good, but the survival rate is significantly lower in anaplastic carcinomas. Although the prognosis of endometrial carcinoma is good, the mortality rate has not decreased due to the increase in the prevalence of the disease and the change in the age composition of the population [9]. The diagnosis of endometrial cancer by pipet biopsy or diagnostic curettage is a method that determines the type of pathology before total abdominal hysterectomy; of course, diagnostic curettage is a better method than pipet. Since 1950, several studies have been conducted to compare the pathological and histological findings of curettage and hysterectomy specimens. According to the obtained results of some of these studies, curettage is an ineffective, risky, and expensive intervention for the diagnosis of endometrial cancer [10][11][12]. In addition, surgical procedures without adequate visualization can lead to errors in diagnosis or complications such as uterine infection, perforation, and cervical laceration [13]. Surgery is often the only way to achieve complete recovery [14]. Sometimes, the pathological results of diagnostic curettage differ from a final total abdominal hysterectomy. Since the type of cancer reported as a result of diagnostic curettage can affect the type of surgery and staging, diagnostic errors in this area can have consequences for the patient's treatment. Staging the disease and planning based on it in the presence of carcinoma are important. For the first stage of endometrial carcinoma, transabdominal hysterectomy with bilateral salpingo-oophorectomy is a selective treatment [15]. Several studies have been conducted to compare the pathological results obtained from curettage with the final pathological results of hysterectomy in patients. The results obtained from some of these studies indicate the high efficiency of curettage in diagnosing endometrial disorders [16][17][18]. Some studies also indicate the inefficiency of curettage in diagnosing endometrial disorders, especially endometrial carcinoma [10][11][12]. In Iran, relatively limited studies have been conducted so far on the pathological comparison of curettage and hysterectomy, and the studies conducted are mostly limited to comparing curettage with other non-invasive sampling methods [19]. For example, according to a study has been conducted in 2006 at Imam Khomeini Hospital in Tehran, curettage was the best diagnostic method for abnormal uterine bleeding, and endometrial biopsy would have a higher diagnostic value if it is accompanied by vaginal ultrasound [20]. However, another study conducted at Mirza Kuchak Khan Hospital in Tehran showed that curettage is an inadequate diagnostic method for focal uterine lesions but has high diagnostic accuracy in endometrial hyperplasia and carcinoma [21]. Early and accurate diagnosis is crucial for guiding treatment decisions, with endometrial curettage being a widely used method for preoperative histopathological assessment [22]. However, studies have reported discrepancies between curettage and final hysterectomy specimens, particularly in grading and depth of invasion assessment, which can affect treatment planning [23]. While previous research has evaluated the diagnostic accuracy of curettage, there is a lack of data from our region, particularly from a high-volume referral center like Shahid Sadoughi Hospital. This study provides a novel five-year retrospective analysis comparing curettage and total abdominal hysterectomy (TAH) pathology results to assess diagnostic concordance. Therefore, the aim of the present study is to compare the clinicopathological results of diagnostic curettage and total abdominal hysterectomy in patients with endometrial cancer referred to Shahid Sadoughi Hospital, Yazd during the years 2019 to 2023.

## Materials and Methods

This study is a cross-sectional analytical study which its statistical population consists of all women with endometrial cancer who underwent diagnostic curettage and total abdominal hysterectomy Their information was recorded in the pathology department of Shahid Sadoughi Hospital in Yazd, Iran from 21.3.2019 to 19.03.2024. The source population was any patients that refer to the hospital, without any limitation on the residential and time duration of the diagnosis. The patients who have any history of prior hormonal therapy, chemotherapy, or radiotherapy before hysterectomy were included. The inclusion criteria in the present study were women diagnosed with endometrial cancer based on histopathological examination, patients who underwent both diagnostic curettage and total abdominal hysterectomy within the study period, patients with available and complete pathology reports from both procedures, age range: ≥30 years (to exclude rare pediatric cases and ensure relevant data), patients with surgically endometrial cancer, without metastasis at the time of hysterectomy and patients who provided informed consent for participation in the study. The incompleteness of the file and information of the studied samples was considered as an exclusion criterion. All samples eligible for inclusion in the study were included in the study at the designated time by census. After the approval of the plan by the research committee of the Department of Obstetrics and Gynecology of Yazd and obtaining approval from the ethics committee of the Faculty of Medicine of Shahid Sadoughi University of Medical Sciences in Yazd and the competent authorities, by referring to the pathology department of Shahid Sadoughi Hospital in Yazd, the desired information was extracted using a structured data collection form, all reports related to curettage and total abdominal hysterectomy samples of patients with endometrial cancer according to the inclusion and exclusion criteria of the study by census method, and confidentially were recorded in Excel file format. All the information required for the study was extracted and recorded based on the research objectives, including demographic information such as age, type of endometrial cancer, clinical manifestations, pathological result of diagnostic curettage, the pathological result of a total abdominal hysterectomy, tumor stage, tumor grade, and lymph node involvement. It should be noted that based on the FIGO criteria [24], tumor grade was divided into two categories: Low grade and High grade. Accordingly, grade 1 endometrioid adenocarcinoma, grade 2 endometrioid adenocarcinoma, were placed in the Low-grade category, and grade 3 endometrioid adenocarcinoma, papillary serous carcinoma, and clear cell carcinoma were placed in the High-grade category.

### Ethical Considerations

The data were analyzed anonymously. All steps of the present study are consistent with the ethical guidelines of the Declaration of Helsinki (World Medical Association for Human Subjects) and were approved by Shahid Sadoughi University of Medical Sciences and Health Services of Yazd (IR.SSU.MEDICINE.REC.2024.234).

### Data Analysis Method

The collected data were statistically analyzed using SPSS V22 (Statistical Package for the Social Sciences, SPSS, Chicago, IL, USA) by computer statistical software. According to the research objectives, the variables were measured using mean ± standard deviation and frequency (percentage) indices; the normality of the data was examined using the 1-Sample K-S test, and considering the non-normal and normal distribution of the data, the statistical tests of Fisher's Exact Test, Chi-Square Tests were used; the significance level was considered equal to 0.05. Furthermore, Sensitivity, Specificity, Positive predictive value (PPV), Negative predictive value (NPV) and Kappa coefficient were obtained.

## Results

**Table:** Table1. The Frequency Distribution of Clinical Manifestations in the Studied Samples

**Variable**	**Frequency**	**Percentage**
**Incidental**	4	4.7
**Vaginal discharge**	2	2.4
**Postmenopausal bleeding**	42	49.4
**Abnormal uterine bleeding**	35	41.2
**Abdominal pain**	2	2.4
**Total**	85	100

Values are reported as number (percentage)

**Table:** Table2. The Frequency Distribution of Curettage Pathology Results in the Studied Samples

**Variable**	**Frequency**	**Percentage**
**Grade 1 Endometrioid adenocarcinoma **	41	48.3
**Grade 2 Endometrioid adenocarcinoma**	8	9.4
**Grade 3 Endometrioid adenocarcinoma**	8	9.4
**Papillary serous carcinoma**	13	15.29
**Clear cell carcinoma**	8	9.4
**Adenocarcinoma or atypical hyperplasia **	7	8.2
**Total**	85	100

Values are reported as number (percentage)

**Table:** Table3. The Frequency Distribution of Hysterectomy Pathology Results in the Studied Samples

**Variable**	**Frequency**	**Percentage**
**Grade 1 Endometrioid adenocarcinoma **	37	43.5
**Grade 2 Endometrioid adenocarcinoma**	15	17.6
**Grade 3 Endometrioid adenocarcinoma**	4	4.7
**Papillary serous carcinoma **	8	9.4
**Progesterone effect**	1	1.2
**Hyperplasia**	3	3.6
**Clear cell carcinoma**	8	9.4
**Normal**	8	9.4
**Atypical hyperplasia complex**	1	1.2
**Total**	85	100

Values are reported as number (percentage)

**Table:** Table4. The Frequency Distribution of Tumor Stage in the Studied Samples

**Variable**	**Frequency**	**Percentage**
**Normal**	11	12.9
**1A**	24	28.2
**3A**	2	2.4
**1B**	23	27.1
**2B**	1	1.2
**3B**	4	4.7
**3C1**	13	15.3
**3C2**	1	1.2
**2**	4	4.7
**4**	1	1.2
**Total**	85	100

Values are reported as number (percentage)

**Table:** Table5. The Comparison of Tumor Grade based on Pathological Findings Obtained from Curettage with Pathological Findings from Hysterectomy in the Studied Samples

**Curettage pathological findings**	**Hysterectomy pathological findings **		**Total**
	**High**	**Low**	
**High**	16 (18. 82%)	11 (12.94%)	27(31.76%)
**Low**	3 (3.53%)	55 (64.71%)	58 (68.24%)
**Total**	19 (22.35%)	66 (77.65%)	85 (100%)
**Sensitivity (%)**			84.21 %
**Specificity (%)**			83.33 %
**Positive predictive value (PPV) (%)**			59.25%
**Negative predictive value (NPV) (%)**			94.82%
**Kappa coefficient**			0.587
**P-value**			0.001

Values are reported as number (percentage)

The mean age of the studied samples was 58.825 ± 11.324 (33-90) years. The highest frequency (47.7%) was 40 in the age range over 60 years and the lowest frequency (21.5%) was 18 in the age range under 50 years. The most common clinical manifestations in participants were postmenopausal bleeding with a frequency of (49.4%) for 42 patients and abnormal uterine bleeding with a frequency of (41.2%) for 35 patients, respectively (Table-1). The most common frequency of curettage pathology results was grade 1 endometrioid adenocarcinoma with a frequency of (48.3%) for 41 patients, papillary serous carcinoma with a frequency of (15.29%) for 13 patients and the least common was adenocarcinoma with a frequency of (8.2%) for 7 patients (Table-2). Also, according to the obtained results, grade 1 endometrioid adenocarcinoma with a frequency of (43.5%) for 37 patients, and grade 2 endometrioid adenocarcinoma with a frequency of (17.6%) for 15 patients, had the most common frequency of pathological findings obtained from hysterectomy, and the least common were atypical hyperplasia complex with a frequency of (1.2%) for 1 patient and progesterone effect with a frequency of (1.2%) for 1 patient (Table-3). Table-4 shows that the highest frequency of tumor stage in the studied samples was 1A with a frequency of (28.2%) for 24 patients, 1B with a frequency of (27.1%) for 23 patients and C13 with a frequency of (15.3%) for 13 patients, respectively. Also, tumor stages 2B and 4 with a frequency of (1.2%) had the lowest frequency. The histopathological results obtained from curettage in terms of grade 7 showed that among of 85 studied samples, 58 cases (68.2%) were from the Low Grade sample and among of 85 studied samples from hysterectomy (77.6%) 66 cases were from the Low Grade sample. As it has shown in Table-5, among of 85 studied samples, the histopathological results (18.82%) for 16 patients of curettage and hysterectomy samples were reported as High Grade (This number is considered as a true positive for curettage). The histopathological results of curettage (12.94%), for 11 participants were reported as High Grade, while the histopathological results of hysterectomy of these individuals were Low Grade (This number is considered as a false positive for curettage). Therefore, the sensitivity, specificity, positive and negative predictive values were calculated for curettage as 84.21%, 83.33%, 59.25% and 94.82%, respectively. Also, the kappa coefficient was reported equal to 0.587. According to the significance of the kappa coefficient, it can be said that the results of curettage and hysterectomy histopathology are consistent with each other (P-value=0.001).

## Discussion

Many researches have been conducted on endometrial cancer and its diagnostic and therapeutic methods in Iran and other countries. By considering the importance of this issue and timely diagnosis and treatment of this disease, the present study was conducted with the aim of comparing the clinicopathological results of diagnostic curettage and total abdominal hysterectomy in patients with endometrial cancer referred to Shahid Sadoughi Hospital in Yazd during the years 2019 to 2023. The results of the present study, which is the result of reviewing the information of 85 files of patients with endometrial cancer with a mean age of 58.825±11.324 (33-90), showed that most people are affected by this disease in the decade 60s their life. In a similar study which was conducted in Urmia city regarding the diagnostic accuracy of curettage and hysterectomy in women with endometrial cancer, it was shown that the highest incidence rate of endometrial carcinoma occurs in the 50s to 60s decades according to the results of hysterectomy in these people [18]. In another study which was conducted on postmenopausal women with vaginal bleeding, the highest incidence of malignancies was related to the 5th and 6th decades of life, respectively [25].

In other studies, it was also reported that the average age of patients with endometrial pathological disorders occurs in the 4th decade of life [20][26]. Therefore, in general, it can be said that the risk of endometrial cancer has increased after the age of 40 years compared to the lower ages than it and is seen more in the Middle Ages. In the present study, the most common clinical symptoms in women were postmenopausal bleeding and abnormal uterine bleeding, respectively, which was similar to the study by Nappi et al., which was conducted on women with atypical endometrial hyperplasia [15][27].

In other studies, the most common clinical symptom was abnormal uterine bleeding [18][26][27], and in the study by Sajitha et al. in India, the most common clinical symptom was menorrhagia [28]. The most common histopathological findings obtained from curettage in the present study were grade 1 endometrioid adenocarcinoma with a frequency of (48.3%) for 41 patients, papillary serous carcinoma with a frequency of (15.29%) for 13 patients and the least common was adenocarcinoma with a frequency of (8.2%) for 7 patients. Also, grade 1 endometrioid adenocarcinoma with a frequency of (43.5%) for 37 patients and grade 2 endometrioid adenocarcinoma with a frequency of (17.6%) for 15 patients had the most common pathological findings obtained from hysterectomy, and the least common was atypical hyperplasia complex with a frequency of (1.2%) for 1 patient and progesterone effect with a frequency of (1.2%) for 1 patient. In a study similar to the present study, the highest frequency of endometrioid carcinoma was (88.63%) for 78 patients [18].

Also, the most common finding in the study by Alshahrani et al., which was conducted from data between 1999 and 2010 in the Western Province of Egypt, was endometrioid adenocarcinoma [29]. In the present study, the highest frequency of tumor stage in the studied samples was1A with a frequency of (28.2%) for 24 patients, 1B with a frequency of (27.1%) for 23 patients and 3C1 with a frequency of (15.3%) for 13 patients, respectively. Also, tumor stages 2B and 4 with a frequency of (1.2%) for 1 patient had the lowest frequency. In the study by Amini Moghadam et al., the highest frequency of tumor stage was 1B with a frequency of (35.48%) for 11 patients and 1A with a frequency of (19.35%) for 6 patients, respectively, which was inconsistent with the present study [3]. In the present study, among 85 samples studied from curettage, 58 samples (68.2%) were Low Grade, and among 85 samples studied from hysterectomy, 66 samples (77.6%) were Low Grade, and the rest of the samples were High Grade. In a study which was conducted in 2022, the patients with endometrioid carcinoma who underwent hysterectomy were reported grade 1 with 66.66%, grade 2 with 15.38% and grade 3 with 10.26% [18].

In a cross- sectional study which was conducted on 31 patients diagnosed with endometrial cancer due to abnormal bleeding by curettage or endometrial biopsy and who were candidates for surgery, 45.16% were grade 1, 19.35% were grade 2, and 35.48% were grade 3 [3]. Diagnosing tumor grade, which is equivalent to the degree of differentiation, can be difficult and depends on the skill of the examiner [30]. In various studies, determining the tumor grading before surgery has a poor relationship with final histologic diagnosis, especially in endometrioid adenocarcinoma tumors that preoperatively their figo grade were 1 and 2, but in grade 3 tumors, the diagnostic concordance between preoperative and postoperative histologic findings was higher [18][31]. In a study which was conducted in 2022 in Thailand, similar to the study by Javanmard et al., the studies showed that the diagnostic accuracy of curettage increases with increasing tumor grade, so that grade 3 tumors had a higher diagnostic accuracy than lower grade tumors.

Therefore, the probability of diagnostic error in curettage is higher in tumors with lower Figo and also in the study by Javanmard et al., the diagnostic accuracy of curettage in grade 3 tumors was 100% and higher than in grade 1 and 2 tumors [18][32]. In the present study, the curettage and hysterectomy specimens (18.82%) for 16 patients were reported as High Grade, but the curettage histopathology results (12.94%) for 11 patients were reported as High Grade, while the hysterectomy histopathology results of these individuals were Low Grade. Therefore, the sensitivity, specificity, positive and negative predictive values calculated for curettage were computed as 84.21%, 83.33%, 59.25% and 94.82%, respectively. The kappa coefficient was also reported equal to 0.587. According to the significance of the kappa coefficient, it can be said that the results obtained from curettage and hysterectomy histopathology are consistent (P-value=0.001). In a study which was conducted in Egypt in 2013, among 83 patients studied, the diagnostic accuracy of curettage was 79% [33]. In another study, the diagnostic accuracy of curettage results in the 5th and 6th decades of life was reported equal to 91.7% and 83.3%, respectively, and it was reported 75% in the 7th decade. Therefore, in general, the diagnostic accuracy of curettage and hysterectomy results was reported more than 70% in people over 50 years. Also, the diagnostic accuracy of curettage in determining tumor type was 78.3%, its sensitivity and specificity were 80.68% and 25%, respectively, and the positive and negative predictive values were 95.95% and 5.65%, respectively [18].

The sensitivity and specificity of curettage in diagnosing endometrial disorders in a study which was conducted in Semnan between the years 2010 and 2015 were 49.1% and 84.5%, respectively, and the positive and negative predictive values were 60.5% and 77.5%, respectively [15]. As it can be seen, in our study, the sensitivity and positive predictive value were higher. However, the low negative predictive value obtained in our study could be influenced by the small number of cases that have had false negative curettage results. In the study by Yarandi et al., the sensitivity and specificity of curettage were 30.2% and 73.3%, respectively, and its positive and negative predictive values were 77.1% and 25.1%, respectively, and the overall accuracy rate was 40.5% which compared with the present study, the sensitivity, specificity, and negative predictive value were lower and the positive predictive value was higher. In this study, they concluded that curettage is an inadequate diagnostic method in focal endometrial disorders, but it is a reliable method in the diagnosis of endometrial hyperplasia and carcinoma [21]. In our study, the results also showed that curettage is a diagnostic method with good accuracy in the diagnosis of endometrial carcinoma. In the study by Hemida et al., the concordance rate between curettage and hysterectomy was 79.5% [33].

In a study which was conducted on 79 patients with endometrial hyperplasia to determine the concordance of histopathological findings before and after hysterectomy, and endometrial specimens obtained by curettage and hysterectomy, the concordance rate for endometrial hyperplasia with both methods was 40.5% .The concordance between pre- and post-hysterectomy findings was not statistically significant (Kappa=0.011).The histopathological findings were more severe after hysterectomy in 6.3% of patients. They concluded that the diagnostic accuracy for endometrial hyperplasia diagnosed by curettage is confirmed [34]. In a study which was conducted in Zahedan in 2017, they introduced piple biopsy as an alternative method for curettage [35], and according to a study which was conducted in Turkey in 2012, they found that there was observed no difference in results between piple biopsy and curettage [36]. In another study which was conducted by Moradan et al., they found the diagnostic value of curettage in the diagnosis of proliferative endometrial disorder and a high diagnostic rate for endometrial cancer with curettage. The results showed that the sensitivity of curettage in the diagnosis of endometrial diseases was 49.1%, the specificity was 84.5%, the positive predictive value was 60.5%, and the negative predictive value was 77.5% [26]. In a retrospective study which was conducted by Bettocchi et al., on 397 patients with abnormal uterine bleeding, in 62.5% of patients, the diagnostic result of curettage was negative but the results obtained of hysterectomy were positive. Therefore, the sensitivity of curettage was 46%, specificity was 100%, positive predictive value was 100%, and negative predictive value was 7.1% [37].

In the present study, for a participant who underwent diagnostic curettage due to abnormal uterine bleeding, the results obtained from histopathology of curettage was grade 1 adenocarcinoma, but the results obtained from histopathology of progesterone-induced hysterectomy was reported. Also, in another participant, the results obtained from histopathology of curettage was atypical hyperplasia, but the histopathology of hysterectomy was reported as simple hyperplasia. In a study which was conducted by Obeidat et al., on 55 patients with abnormal uterine bleeding, it was found that 47.3% of participants had simple hyperplasia, 43.6% had mixed hyperplasia, and 9.1% had atypical mixed hyperplasia. The concordance rate between histopathological findings in curettage and hysterectomy specimens was 45.5%. By performing hysterectomy, none of the 26 patients with simple hyperplasia had endometrial cancer, but one patient with cancer was diagnosed with concurrent hyperplasia. Therefore, it was concluded that curettage is consistent with histopathological findings in simple hyperplasia disease, but curettage appears to have less diagnostic value in mixed atypical hyperplasia [38].

In another study, it was observed that endometrial curettage results in patients with simple and complex cystic atypical hyperplasia is consistent with hysterectomy results, but curettage appears to have less diagnostic value in patients with adenomatous hyperplasia. Furthermore, in general, the sensitivity of diagnosis of endometrial hyperplasia by curettage is low (62.5%). The results of this study showed that although endometrial cancer was not diagnosed in any of the patients with adenomatous hyperplasia and simple cystic hyperplasia, but endometrial carcinoma was diagnosed in 2 patients with abnormal complex hyperplasia before hysterectomy [33].

Also, in the study by Kurt et al., in 58 patients with abnormal endometrial hyperplasia, the results showed that although endometrial cancer was not diagnosed in any patient with abnormal endometrial hyperplasia before hysterectomy, but differentiated endometrial adenocarcinoma was identified in 44.7% of the patients [39].

Among the limitations of the present study, the analysis is based on cross-sectional data; therefore, causal inference cannot be extracted. Although the present study was conducted with a larger sample size than other similar studies, considering that some of the studied variables were not significant, contrary to the possible hypotheses; it is recommended that this study should be conducted in a multi-center setting with a larger sample size for a more accurate study. It is also recommended that in the future studies, the diagnostic accuracy of curettage in each endometrial disease should be examined.

## Conclusion

The sensitivity and specificity of curettage suggest a high level of accuracy in comparison with total abdominal hysterectomy findings, with a high value of NPV. However, PPV of the curettage suggests a moderate level in comparison with the total abdominal hysterectomy. Moreover, the kappa coefficient further supports a moderate agreement between curettage and hysterectomy pathology.

Therefore, in the present study it is concluded that the diagnostic accuracy of curettage compared to total hysterectomy is a relatively suitable diagnostic method for evaluating endometrial cancer, this method may be used in the timely diagnosis of this disease. However, caution is required due to the lower PPV, necessitating further evaluation before definitive management.

## Conflict of Interest

The authors had no conflict of interest in conducting the present study.
